# Novel NF-κB reporter mouse for the non-invasive monitoring of inflammatory diseases

**DOI:** 10.1038/s41598-023-29689-4

**Published:** 2023-03-02

**Authors:** Se Yong Park, Min Woo Kim, Ju-Hee Kang, Hyun Jin Jung, Jung Ho Hwang, Soo Jung Yang, Jong Kyu Woo, Yoon Jeon, Ho Lee, Yeo Sung Yoon, Je Kyung Seong, Seung Hyun Oh

**Affiliations:** 1 College of Veterinary Medicine, Seoul National University, Seoul, Republic of Korea; 2 College of Veterinary Medicine, Konkuk University, Seoul, Republic of Korea; 3 College of Pharmacy, Gachon University, Incheon, Republic of Korea; 4 Korea Mouse Phenotyping Center (KMPC), College of Veterinary Medicine, Seoul National University, Seoul, Republic of Korea; 5 Graduate School of Cancer Science and Policy, Research Institute, National Cancer Center, Goyang, Republic of Korea

**Keywords:** Imaging, Inflammation

## Abstract

Bioluminescence imaging is useful for non-invasively monitoring inflammatory reactions associated with disease progression, and since NF-κB is a pivotal transcription factor that alters expressions of inflammatory genes, we generated novel NF-κB luciferase reporter (NF-κB-Luc) mice to understand the dynamics of inflammatory responses in whole body, and also in various type of cells by crossing NF-κB-Luc mice with cell-type specific Cre expressing mice (NF-κB-Luc:[Cre]). Bioluminescence intensity was significantly increased in NF-κB-Luc (NKL) mice exposed to inflammatory stimuli (PMA or LPS). Crossing NF-κB-Luc mice with Alb-cre mice or Lyz-cre mice generated NF-κB-Luc:Alb (NKLA) and NF-κB-Luc:Lyz2 (NKLL) mice, respectively. NKLA and NKLL mice showed enhanced bioluminescence in liver and macrophages, respectively. To confirm that our reporter mice could be utilized for the non-invasive monitoring of inflammation in preclinical models, we conducted a DSS-induced colitis model and a CDAHFD-induced NASH model in our reporter mice. In both models, our reporter mice reflected the development of these diseases over time. In conclusion, we believe that our novel reporter mouse can be utilized as a non-invasive monitoring platform for inflammatory diseases.

## Introduction

Inflammation is an essential body defense and necessary for healing^1^, but excessive and uncontrollable inflammation is a major pathogenic feature of inflammatory diseases^2^. As a result, inflammation is a therapeutic target, and many researchers and pharmaceutical companies have tried to understand the mechanism of the pathogeneses of inflammatory diseases and develop effective anti-inflammatory drugs with few side effects.

NF-κB (Nuclear factor kappa-light-chain-enhancer of activated B cells) is a pivotal transcription factor and regulator of inflammatory and immune responses. When activated, NF-κB complex translocates to the nucleus, where it induces the expressions of proinflammatory genes including those of cytokines and chemokines^3^. Previous studies have reported the involvement of activated NF-κB pathway in various inflammatory diseases, such as non-alcoholic steatohepatitis (NASH)^4^, inflammatory bowel disease (IBD)^5^, rheumatoid arthritis^6^, atherosclerosis^7^, and multiple sclerosis^8^, and thus, NF-κB is viewed as a propitious therapeutic target in inflammatory diseases^9^. Also, NF-κB activation is commonly used experimentally to evaluate inflammation in vitro and in vivo.

Usually, laboratory animals used for inflammation research and inflammation evaluation are sacrificed, and studies are performed postmortem on tissue and blood samples^10^. However, unless many animals are used to obtain results at multiple time points this type of analysis cannot reflect the dynamics of longitudinal inflammatory responses^11^. To overcome this limitation, In vivo live imaging techniques including optical imaging, magnetic resonance imaging, and computed tomography have been developed. In particular, optical imaging has been widely used in the live imaging field due to its sensitivity, safety, ease of use, and cost-effectiveness^12^.

In vivo bioluminescence imaging (BLI) is one such optical imaging modality and is based on the detection of luminescence signals in living organisms expressing the luciferase reporter gene^13^. Luciferase is an oxidative enzyme that produces bioluminescence in the presence of its substrate, D-luciferin. During this enzyme–substrate reaction, luciferase oxidizes D-luciferin and some of the energy produced is emitted as light, which can be quantified using a charge-coupled device (CCD) to yield information on biological processes^14^. As compared with fluorescence, luciferase signal has higher signal to background noise ratio than fluorescent which feature make bioluminescence more suitable for detection of weaker signals from internal organs^15^. Above all, in vivo BLI allows longitudinal monitoring without sacrifice^11^ and provides insights into complex and dynamic phenomena, while postmortem analysis provides only cross-sectional information.

In vivo BLI has been used for various purposes, such as for luciferase-labeled exogenous cell tracking^16^, infection evaluations^17^, and gene activity reporting^18^. NF-κB luciferase reporter mouse models have also been reported^19,20^, but these reporter mice expressed luciferase in all cells when NF-κB was activated. However, inflammation is a complicated process, and each inflammatory disease has a unique pathogenesis that involves different cell types^21^. Whole body and specific cell inflammation assessments are crucial for studying inflammatory diseases and understanding their mechanisms.

In this study, we generated a novel NF-κB luciferase reporter mouse in a C57BL/6 background (NF-κB-Luc) which can be further utilized as cell-type specific enhanced luciferase reporter mouse (NF-κB-Luc:[Cre]) after crossed with cell-type specific Cre recombinase expressing mice to investigate the dynamics of inflammatory responses in aspect of specific cell-type as well as whole body.

## Materials and methods

### Generation of NF-κB luciferase reporter mice

The NF-κB reporter construct contained 4 repeats of NF-κB responsive element (5'-GGGAATTTCC-3'), minimal TA promoter (5'-TAGAGGGTATATAATGGAAGCTCGAATTCCAG-3'), Luciferase reporter gene, ubiquitin C (UBC) promoter, and the tdTomato fluorescence gene. Minimal TA promoter includes the TATA box from Herpes simplex virus thymidine kinase (HSV-tk) promoter. The firefly luciferase gene was amplified from NF-κB-luciferase plasmid (Clontech, CA, USA). To produce the targeting vector, the NF-κB reporter construct was cloned into the FseI site of Ai9 vector^22^. The targeting vector was linearized with KpnI and electroporated into mouse embryonic stem cells (mES)^23^, and 192 mES cell colonies that are tdTomato-positive and neomycin-resistance were picked. Chimeric males were bred with C57BL/6 females and the heterozygotes were backcrossed to C57BL/6 for at least 6 generations.

### Animal care, breeding, and genotyping

Mice were maintained under a 12 h light/12 h dark cycle at 22 °C and 60% RH in a specific pathogen-free facility at Gachon University. All animal experiments were approved by the Institutional Animal Care and Usage Committee (IACUC) at Gachon University (approval no. GU1-2021-IA0037) and carried out in accordance with the committee’s guidelines, and the study was performed according to ARRIVE guidelines^24^. Genotyping was performed using tail genomic DNA. The primer sequences used for genotyping are provided in Supplementary Information. All in vivo experiments were conducted using 8- to 10-week-old mice. At the end of each in vivo experiment, all mice were euthanized by isoflurane inhalation.

### Bioluminescence imaging

Bioluminescence was detected under isoflurane inhalation anesthesia. After anesthesia induction, each mouse was intraperitoneally (i.p.) injected with d-luciferin (100 µl, 15 mg/ml in PBS) (GoldBio, MO, USA) 5 min before measurements and signals were obtained for 10 s using an Ami X (Spectral Instruments Imaging, Ontario, Canada) or VISQUE InVivo ART100 (Vieworks Co., Ltd., Anyang-si, Korea), and data obtained were analyzed using the Aura or CleVue programs, respectively. ROIs were drawn on ear or abdominal region depending on the target organ of each in vivo experiments.

### The phorbol 12-myristate 13-acetate (PMA)-induced ear edema model

C57BL/6 wildtype mice (n = 6) and NF-κB-Luc mice (n = 6) were topically treated with 2 µg of PMA (Sigma-Aldrich, MO, USA) on right ears And with ethanol on left ears (vehicle controls). Six hours later, three mice in each group were injected with D-luciferin i.p., and bioluminescence was measured. After measurements, right and left ears were harvested for histopathological analysis.

### The lipopolysaccharide (LPS)-induced systemic inflammation model

NF-κB-Luc mice (n = 9) were randomly divided into two groups, that is, the PBS group (n = 3) and the LPS group (n = 6). PBS was injected into mice in the PBS group, while mice in the LPS group were injected with LPS (Sigma-Aldrich) at 5 mg/kg i.p. Bioluminescence signals were measured at three time points (before injection, 2 h after injection, and 6 h after injection). Mice in the PBS group were sacrificed at 6 h after injection. The LPS group was further divided into two groups (n = 3 per group), and animals in these groups were sacrificed at 2 or 6 h after LPS injection. At necropsy, blood was collected through cardiac puncture, and serum was isolated for inflammatory cytokine analysis. Livers, lungs, and kidneys were harvested and frozen for western blotting.

### The dextran sodium sulfate (DSS)-induced acute colitis model

NF-κB-Luc:Lyz2 mice (n = 3) were treated with 3% (w/v) DSS (36–50 kDa) (MP Biomedicals, OH, USA) in drinking water for 5 days. Bioluminescence was measured at baseline and at 1, 3, and 5 days after treatment commencement. Body weights and disease activity indices (DAIs) were measured before bioluminescence intensities. DAI scores were defined as the sum of stool consistency scores (0–3) and blood in stool scores (0–3). On day 5, all mice were sacrificed under anesthesia, and colons were harvested for histopathological analysis. At necropsy, an age-matched NF-κB-Luc:Lyz2 mouse was sacrificed and its colon was harvested as a control.

### The choline-deficient, l-amino acid-defined, high-fat diet (CDAHFD)-induced NASH model

NF-κB-Luc mice (n = 9) and same-aged NF-κB-Luc:Alb mice (n = 9) were each divided into three groups (n = 3 per group), which were fed a normal chow diet (Woojungbio, Hwasung, Korea) for 2 weeks or a CDAHFD (Research Diets Inc., NJ, USA) for 1 or 2 weeks. Body weights of mice fed the CDAHFD for 2 weeks were measured every 3–4 days during experiments. Bioluminescence intensities were measured on completing these feeding schedules. All mice were then sacrificed, blood was collected through cardiac puncture and livers were harvested for histopathological analysis. Serum levels of aspartate aminotransferase (AST) and alanine aminotransferase (ALT) were measured using a HITACHI 7180 autoanalyzer (Hitachi, Ltd., Tokyo, Japan).

### Histopathological analysis

Harvested tissues were fixed with 10% neutral buffered formalin and embedded in paraffin blocks, sectioned at 5 μm, hematoxylin–eosin (HE) stained, and observed under an optical microscope. Histopathological changes in the liver sections of the CDAHFD-induced NASH model were evaluated using non-alcoholic fatty liver disease (NAFLD) activity scores (NAS) as previously described^25^.

### Enzyme-linked immunosorbent assay (ELISA)

Serum inflammatory cytokines, interleukin 1 beta (IL-1β), interleukin 6 (IL-6), and murine tumor necrosis factor alpha (TNFα), were detected using a commercially available ELISA kits (R&D Systems, MN, USA), according to the manufacturer’s instructions. Serum samples were diluted tenfold using the diluent included in the ELISA kit.

### Primary cell isolation and culture

Primary macrophages, peritoneal macrophages and bone-marrow derived macrophages (BMDM), were isolated from NF-κB-Luc (NKL), NF-κB-Luc:Lyz2 (NKLL), and NF-κB-Luc:Alb (NKLA) mice, as previously described^26^, and primary mouse hepatocytes were isolated from NKL and NKLA mice as previously described^27^ with slight modification. Detailed methods are described in Supplementary Information.

### Western blotting

Western blotting was carried out as previously described^28^. Cells and tissues were lysed with modified RIPA buffer, and total proteins were extracted and quantified. Same amounts of total proteins were separated by SDS-PAGE and transferred to PVDF membranes, which were blocked with 5% (w/v) skim milk in tris buffer saline containing 0.1% Tween 20 (TBS-T). After washed, the membranes were incubated with primary antibodies, and then incubated with appropriate secondary antibodies. Immunoreactive bands were detected using Absignal™ western blot detection kit (Abclon, Seoul). The developed films were cut to each PVDF membrane size and scanned to generate western blot images. Those images were cropped for presenting target proteins in the figures. Original images of scanned films are presented in Supplementary Information. Primary antibodies used for western blotting were Luciferase (Sigma-Aldrich), GAPDH (Merck Millipore, Darmstadt, Germany), and Actin (Santa Cruz Biotechnology, CA, USA).

### In vitro luminescence measurement

Primary hepatocytes were seeded on white 96-well plates and treated with TNFα (Peprotech, NJ, USA) or BAY 11-7085 (Cayman Chemical Co, MI, USA). After indicated times, media were replaced with 1X Hank's balanced salt solution (HBSS) (Welgene) containing D-luciferin at 150 µg/ml, and luminescence intensities were measured using VICTOR X3 plate reader (PerkinElmer, MA, USA).

### Statistical analysis

The analysis was conducted using the Student's t-test, and results are presented as means ± standard errors of means (SEMs). Statistical significance was accepted for P values < 0.05.

## Results

### Generation of NF-κB luciferase reporter mice

**Figure 1:**
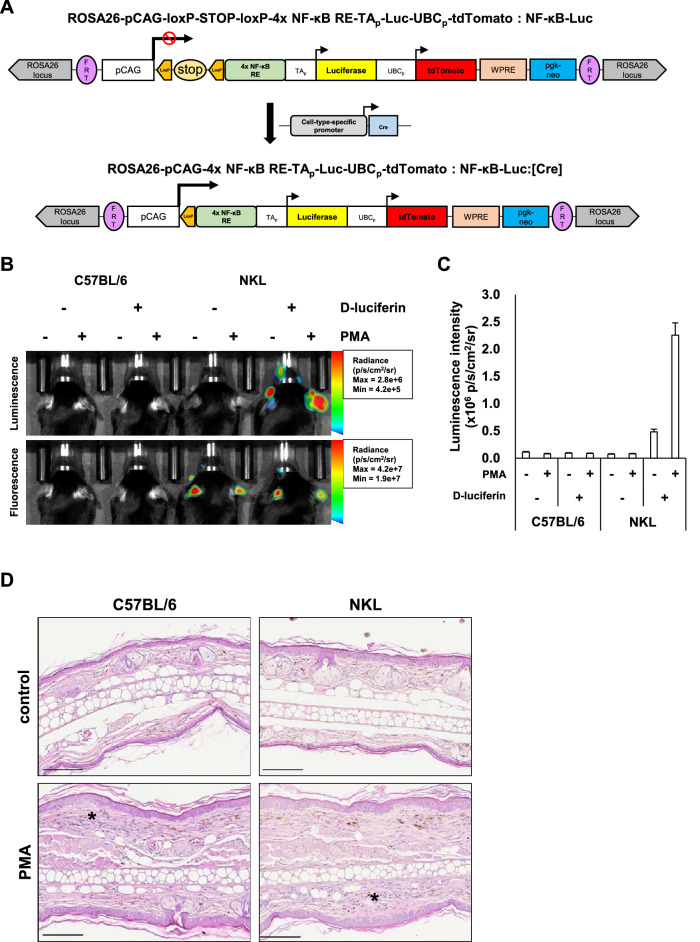
Generation of NF-κB luciferase reporter mice. (**A**) Construct of the NF-κB luciferase reporter gene cassette integrated into the ROSA26 locus. pCAG, synthetic promoter consisting of CMV enhancer fused to the chicken beta-actin promoter; RE (responsive element), four repeats of NF-κB binding sequence; Stop, three repeats of SV40 polyA; TAp, minimal TA promoter (including the TATA box from HSV-tk promoter); WRPE, the woodchuck hepatitis virus posttranscriptional regulatory element; pgk-neo, neomycin-resistant gene driven by pgk promoter. (**B**) Bioluminescence and fluorescence images of C57BL/6 wild-type mice and NF-κB-Luc mice (NKL) in the PMA-induced ear inflammation model. PMA (2 μg) was applied to the right ears, and 6 h later, bioluminescence and fluorescence signals were detected 5 min after D-luciferin (100 µl, 15 mg/ml in PBS) i.p. injection. Radiance ranges of parameters are provided in the figure. (**C**) Mean group luminescence intensities (n = 3 per group). Results are presented as means ± SEMs. (**D**) Representative images of HE stained vehicle or PMA-treated ears (scale bar = 100 μm). Asterisk (*); thickened dermis with infiltrated immune cells.

Bioluminescence has been applied in preclinical studies since it allows investigators to visualize disease-relevant in vivo events and reduces the number of mice utilized for in vivo spatiotemporal analyses^29^. In this study, we generated a novel NF-κB luciferase reporter mouse line to non-invasively monitor the dynamics of whole body and specific cell inflammation (Fig. 1A). The luciferase reporter gene cassette was inserted into ROSA26 allele to evaluate NF-κB activation levels ubiquitously (NF-κB-Luc), and the tdTomato fluorescence reporter gene under the control of ubiquitin C promoter was used as a selection marker. After crossed with cell-type specific Cre recombinase expressing mouse, luciferase activity following NF-κB activation will be enhanced in that specific cell type through the intervention of CMV early enhancer/chicken β-actin (CAG) promoter (NF-κB-Luc:[Cre]). Since PMA can induce inflammatory reactions via NF-κB activation^30^, we used a PMA-induced ear inflammation model as a representative of inflammatory diseases in superficial tissues to determine whether bioluminescence can be induced by inflammatory stimuli. Regardless of PMA treatment, NF-κB-Luc (NKL) mice showed tdTomato fluorescence in hairless regions (ears and noses) versus wild-type C57BL/6 mice (WT mice) (Fig. 1B). WT mice did not produce any bioluminescence signals after D-luciferin injection, but bioluminescence signals were detected from D-luciferin injected NKL mice, and the intensities of these signals were much higher in PMA-treated ears than vehicle-treated ears in same mice (Fig. 1B,C). To confirm the presence of PMA-induced ear inflammation, vehicle and PMA-treated ears were sectioned and HE stained. Though luminescence intensities differed, PMA induced histopathological changes in ear thicknesses and immune cells infiltrations were similar between WT mice and NKL mice (Fig. 1D). These results showed our reporter mouse reflected inflammation status as luminescence intensity after applying topical inflammatory stimulus.

### Non-invasive tracking of lipopolysaccharide (LPS)-induced inflammation

**Figure 2:**
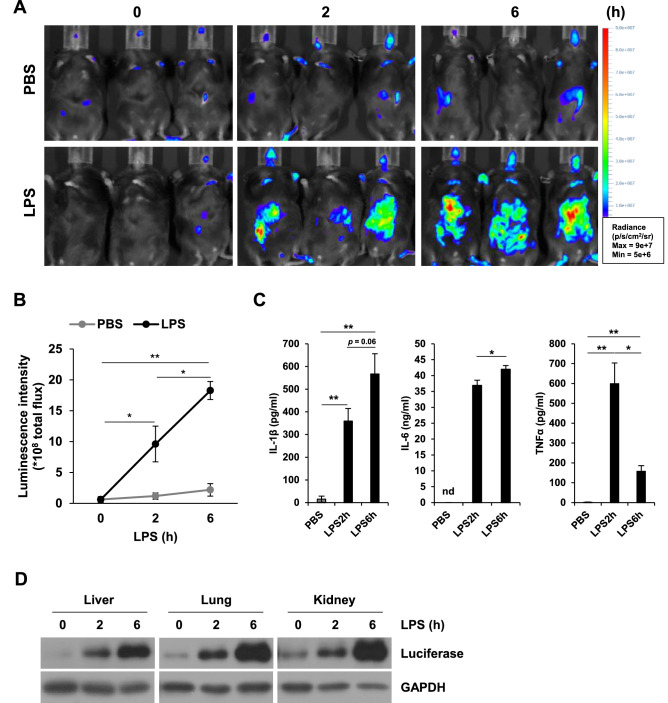
LPS-induced systemic inflammation applied to NF-κB-Luc mice. (**A**,**B**) Bioluminescence images of PBS or LPS injected NF-κB-Luc mice (**A**) and total abdominal luminescence fluxes (**B**). The images were obtained at different times from each group (n = 3) using VISQUE InVivo ART100. Radiance ranges are provided in the figure. (**C**) Serum levels of IL-1β, IL-6, and TNFα. Results are presented as means ± SEMs (n = 3). *p < 0.05, **p < 0.01, *nd* not detected. (**D**) Representative expressions of luciferase in liver, lung, and kidney as determined by western blotting. All western blot images were cropped from the original films presented in Supplementary Information.

The LPS-induced systemic inflammation mouse model developed to mimic human sepsis showed exogenous LPS injection elicits inflammatory responses via NF-κB activation^31^. Thus, we subjected NKL mice to LPS-induced systemic inflammation to evaluate whether our reporter mice were sufficiently sensitive to detect NF-κB activation in internal organs. LPS injected NKL mice showed stronger luminescence in the abdominal region than PBS injected NKL mice (Fig. 2A), and this luminescence was significantly increased over time after LPS injection (Fig. 2B). Elevated serum levels of IL-1β, TNF-α, and IL-6 which are well-known as pro-inflammatory cytokines confirmed that this increase in luminescence was due to LPS-induced systemic inflammation (Fig. 2C). Furthermore, the levels of luciferase in livers, lungs, and kidneys were increased by LPS treatment, which confirmed these inflammatory responses were due to luminescence emanating from internal organs (Fig. 2D). These findings showed that our reporter mouse provides a useful means of monitoring inflammatory reaction from deep internal organs.

### Generation of cell-type specific enhanced reporter mice and ex vivo applications

**Figure 3:**
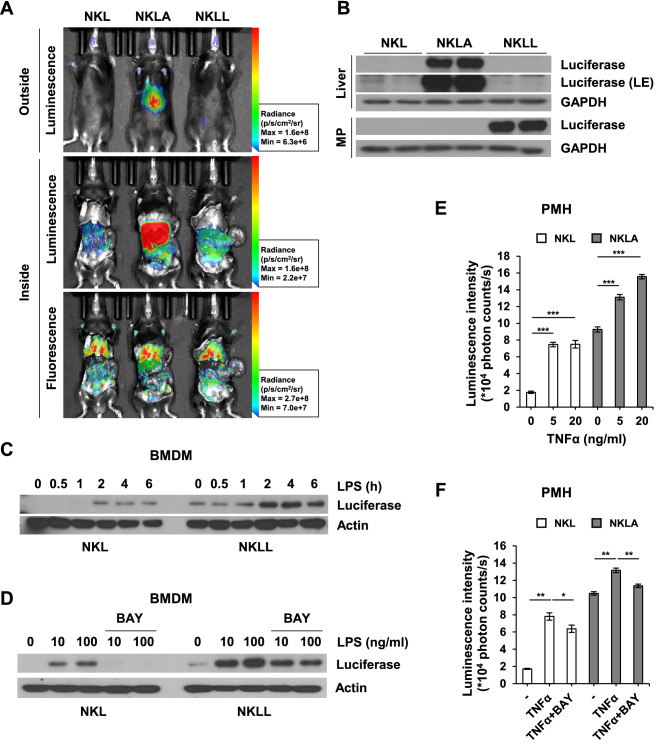
Generation of cell-type specific enhanced reporter mice and its ex vivo applications. (**A**) Comparison of bioluminescence and fluorescence among genotypes. Radiance ranges of parameters are provided in the figure. (**B**) Luciferase levels in liver and peritoneal macrophages isolated from NF-κB-Luc mice (NKL), NF-κB-Luc:Alb mice (NKLA), and NF-κB-Luc:Lyz2 mice (NKLL). LE; long exposure. (**C**,**D**) Luciferase levels in BMDMs isolated from NKL or NKLL. BMDMs were treated with LPS (100 ng/ml) for different times (**C**) or co-treated with LPS (100 ng/ml) and BAY 11–7085 (10 μM) for 6 h (**D**). Protein expressions were detected by western blotting. (**E**,**F**) Luminescence intensities of primary mouse hepatocytes (PMHs) isolated from NKL or NKLA. PMHs were treated with TNFα (5 or 20 ng/ml) (**E**), or co-treated with TNFα (20 ng/ml) and BAY 11–7085 (10 μM) for 24 h (**F**). Luminescence intensities were measured using microplate reader, and results are presented as means ± SEMs (n = 5). *p < 0.05, **p < 0.01, ***p < 0.001. *MP* peritoneal macrophage, *BMDM* bone marrow-derived macrophage, *PMH* primary mouse hepatocyte. All western blot images were cropped from the original films presented in Supplementary Information.

Beyond NKL mice itself can be utilized as a reporter mouse for NF-κB activation in whole body at low signal, NKL mice also used as a cell-type specific enhanced NF-κB luciferase reporter mouse lines (NF-κB-Luc:[Cre]) when crossed with Cre recombinase expressing mice. In this study, we produced two NF-κB-Luc:[Cre] mouse lines by crossing NF-κB-Luc mice with Alb-cre or Lyz2-cre mice to generate hepatocyte-specific (NF-κB-Luc:Alb ; NKLA) or myeloid cell-specific (NF-κB-Luc:Lyz2 ; NKLL) enhanced NF-κB luciferase reporter mouse lines, respectively. NKLA mouse showed enhanced luciferase activity in livers than NF-κB-Luc mouse, whereas NKLL mouse showed enhanced luciferase activity in peritoneal organs, where peritoneal macrophages reside (Fig. 3A). However, fluorescence intensities were similar among genotypes (Fig. 3A). As observed in bioluminescence images, luciferase levels were enhanced in the liver tissues of NKLA mice and in the peritoneal macrophages of NKLL mice (Fig. 3B). In addition, our reporter mouse is a useful source of primary cells containing a luciferase reporting system that responds to NF-κB activation. Luciferase levels were increased by LPS treatment in BMDMs isolated from NKL mice and NKLL mice, but higher in BMDMs from NKLL mice than those from NKL mice (Fig. 3C). Furthermore, luciferase levels were time- and dose-dependently increased by LPS treatment, and these increases were suppressed by BAY 11–7085, an NF-κB inhibitor (Fig. 3C,D). Consistently, primary hepatocytes isolated from NKLA mice showed higher luminescence intensities than primary hepatocytes isolated from NKL mice, and these luminescence intensities were increased by TNFα (Fig. 3E). Also, TNFα-induced increases of luminescence intensities in primary hepatocytes were downregulated by BAY 11-7085 (Fig. 3F). Taken together, our reporter mice exhibited whole body (NF-κB-Luc) and cell-type specific NF-κB dependent bioluminescence (NF-κB-Luc:[Cre]).

### Non-invasive monitoring of inflammatory signals in the IBD model

**Figure 4:**
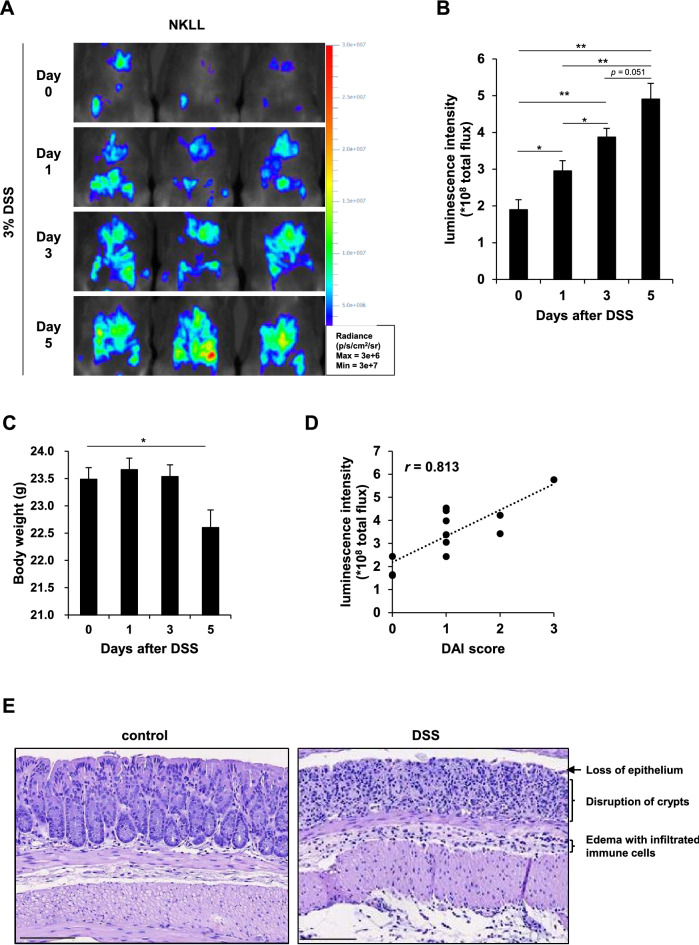
Non-invasive monitoring of inflammatory signals in the IBD model. NF-κB-Luc:Lyz2 (NKLL) mice were treated with 3% (w/v) dextran sodium sulfate (DSS) in drinking water for 5 days (n = 3) (**A**) Abdominal bioluminescence images and (**B**) luminescence intensities. Radiance ranges are provided in the figure. Luminescence intensity increased time-dependently after DSS treatment. (**C**) Body weight change during DSS treatment. (**D**) Correlation between DAI scores and luminescence intensities. The correlation coefficient (*r*) is shown in the figure. (**E**) Representative images of HE staining of colon tissues from mice before (control) and after 5 days of DSS treatment (DSS) (Scale bar = 100 μm). Histopathological features are indicated in the figure. *p < 0.05, **p < 0.01.

Inflammatory bowel diseases (IBD), which includes Crohn’s disease and ulcerative colitis, is a global health issue, and thus, there is an urgent need to elucidate the molecular mechanisms responsible and develop effective drugs. Several animal models have been used in preclinical studies to evaluate the IBD targeting efficacies of candidate drugs. Of the preclinical animal models available, dextran sodium sulfate (DSS)-induced colitis mouse model is the most widely used due to its rapidity, simplicity, reproducibility, and controllability^32^. Since NF-κB is activated in macrophages during the development of IBD^5^, NKLL mice, which exhibit enhanced bioluminescence in macrophages, were treated with DSS to induce acute colitis to assess whether these reporter mice reflect myeloid-cell dependent inflammatory reactions. As was expected, abdominal bioluminescence was time-dependently increased by 3% (w/v) DSS (Fig. 4A). Furthermore, luminescence intensities were significantly increased after 24 h of DSS treatment while body weights were unaffected (Fig. 4B,C). Also, bioluminescence intensities were strongly correlated with disease activity scores (DAIs) (Fig. 4D). Histopathologically, colons of DSS-treated mice showed changes typically associated with colitis, such as loss of epithelium, disruption of crypt structure, and infiltration of immune cells in mucosa and submucosa (Fig. 4E). These results suggest that our reporter mice could be used to non-invasively monitor inflammatory reactions from the early stages of colitis development.

### Non-invasive evaluation of inflammation in the NASH model

**Figure 5:**
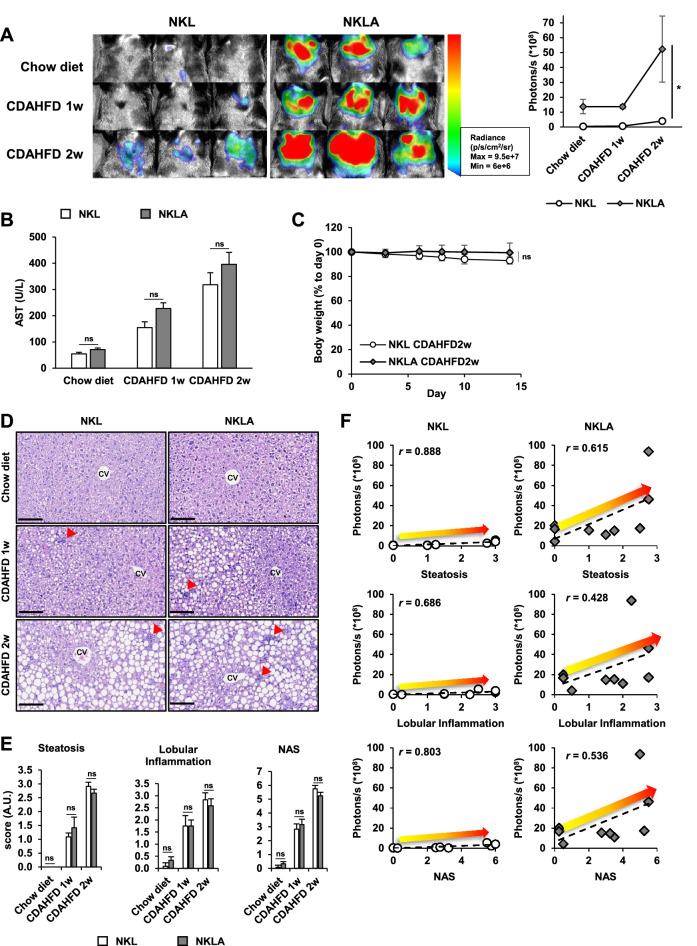
Non-invasive monitoring of inflammatory signals in the NASH model. NF-κB-Luc (NKL) and NF-κB-Luc:Alb (NKLA) mice were fed normal chow diet for 2 weeks or choline-deficient, L-amino acid-defined, high-fat diet (CDAHFD) for 1 or 2 weeks. (n = 3 per group) (**A**) Bioluminescence images of Chow diet group, CDAHFD 1w group, and CDAHFD 2w group for both genotype. The images were obtained at the end of experiments, and the bioluminescence intensities were represented as a graph. The experimental schedule is described in detail in Materials and Methods. (**B**) Serum aspartate aminotransferase (AST) levels and (**C**) body weight changes of NKL and NKLA mice. (**D**) Representative HE images of livers obtained from each group of NKL and NKLA mice (scale bar = 100 μm), and (**E**) histopathological scores. cv; central vein, arrowhead; inflammatory foci. (**F**) Correlations between histopathological scores and luminescence intensities. Correlation coefficients (*r*) are shown in the graphs. *ns* not significant.

The prevalence of obesity-related inflammatory diseases, particularly NASH, have increased in parallel with a worldwide increase in obesity^33^. Unfortunately, no drug has been FDA approved for the treatment of NASH despite the efforts of many researchers and pharmaceutical companies. However, genetically developed NASH and diet-induced NASH have been established for preclinical studies, and of these models, the CDAHFD (choline-deficient, l-amino acid-defined, high-fat diet)-induced NASH model, which also induces fibrosis, is considered rapid and reproducible^34^. Actually, even 1 week of CDAHFD treatment can induce NASH in mice^35^. Since the NF-κB signaling pathway is one of the major regulators of liver inflammation and NF-κB is activated during the development NASH^4,36^, we treated NKL and NKLA mice with CDAHFD to determine whether our reporter mice could reflect hepatic inflammation. Bioluminescence in livers was higher after CDAHFD treatment that in those of normal chow diet controls for both genotypes, but bioluminescence intensities were several times higher in NKLA mice than in NKL mice (Fig. 5A). On the other hand, serum AST levels (Fig. 5B) and body weight changes were non-significantly different between genotypes (Fig. 5C), indicating that the development of NASH was not dependent on genotype. In addition, though HE staining of liver tissues showed lipid accumulation in hepatocytes and immune cell infiltration increased over time after CDAHFD treatment, no differences were observed between genotypes (Fig. 5D). Consistently, histopathological scores were increased by CDAHFD, but no genotype differences were observed (Fig. 5E). Furthermore, for both genotypes, bioluminescence intensities and histological scores of liver tissues were positively correlated (Fig. 5F). These results show that the devised mouse reporter system can be used to monitor inflammatory status non-invasively NASH development.

## Discussion

Inflammatory diseases have complicated pathologies and different mechanisms, and thus detailed experimental models are essential to understand inflammatory diseases and develop anti-inflammatory drugs. Usually, laboratory animal models are used to study inflammatory diseases, and postmortem analysis is used to evaluate inflammation severities^10^. However, although postmortem analysis provides valuable information on inflammatory states, it does not provide information on dynamic changes in inflammation. To overcome this shortcoming, BLI has been widely used to non-invasively monitor inflammatory reactions associated with disease progression.

Since NF-κB is one of the substantial transcription factors that alter the expressions of inflammatory genes, NF-κB driven luciferase reporter systems have been used to detect inflammatory signals. Previous studies have described in vivo NF-κB reporter models based on NF-κB-driven luciferase^19,20^, but these models are based on whole body NF-κB activation. However, inflammatory diseases involve different key cell types^21^, and thus, cell-type specific assessments of inflammation are required to understand these diseases comprehensively and aid target-specific drug development.

In this study, we generated a novel NF-κB driven luciferase reporter mouse which can be utilized as a reporter system reflecting inflammatory signals in whole animals or specific cell types by combining NF-κB luciferase reporter system and Cre-loxP system (Fig. 1A). The reporter gene cassette was inserted into the ROSA26 locus to enable stable expression and minimize unpredictable insertional effects. Also, based on considerations of compatibility with previously established mouse resources, we utilized C57BL/6 mice as a background, as they have been frequently used to establish Cre recombinase lines and knock-out lines^37,38^. Since the tdTomato reporter gene was used as an embryo selection marker when we produced reporter mice, NKL mice emanated tdTomato fluorescence signals, while C57BL/6 wild-type mice (WT mice) did not (Fig. 1B). To evaluate whether NKL mice can reflect inflammation, we used a PMA-induced ear inflammation model as a representative model of inflammatory diseases of superficial organs. Bioluminescence was highly detected only from the PMA-treated ears of D-luciferin injected NKL mice (Fig. 1B,C), while ear inflammation was similarly induced by PMA in WT mice and NKL mice (Fig. 1D). These results confirmed that NKL mice can be used to monitor inflammatory signals from superficial organs and do not exhibit pathophysiological differences versus WT mice.

The LPS-induced systemic inflammation model was performed to evaluate inflammatory signals originating from internal organs of our reporter mice. As was expected, inflammation was induced by LPS injection and was observed as an increase in bioluminescent signals (Fig. 2A,B). In addition, serum IL-1β, IL-6, and TNFα levels were elevated in our LPS-induced systemic inflammation model (Fig. 2C), which is consistent with the induction of inflammatory cytokines by LPS through a toll-like receptor 4 signaling pathway^39^. Moreover, we confirmed that the observed BLI (Fig. 2A) was caused by the induction of luciferase expression (Fig. 2D). These results suggest that our reporter mouse could be used to detect inflammatory signals from internal organs in vivo.

We also produced cell-type specific NF-κB driven luciferase reporter mice, NKLA and NKLL, by crossing NKL mice with Alb-cre and Lyz2-cre mice, respectively. NKLA mice showed enhanced bioluminescence in liver due to hepatocyte specific enhancement of luciferase expression, whereas NKLL mice showed enhanced bioluminescence from the abdominal cavity where peritoneal macrophages reside (Fig. 3A). Similarly, luciferase levels were enhanced in liver and peritoneal macrophages from NKLA and NKLL mice, respectively (Fig. 3B). We also isolated BMDMs and primary hepatocytes and stimulated them with LPS or TNFα to determine whether the reporter system sufficiently reflected inflammatory status ex vivo. Luciferase expression was induced by LPS in BMDMs, and luminescence intensities in primary hepatocytes were increased by TNFα treatment (Fig. 3C,E). In addition, increases in luciferase levels or bioluminescence were down-regulated by BAY 11-7085, an NF-κB inhibitor, which indicated these increases were due to NF-κB activation (Fig. 3D,F). These ex vivo results showed that the devised reporter mouse system can be used as a source of primary cells, and that primary cells containing the NF-κB reporter system can be utilized for screening anti-inflammatory drugs.

To validate the suitability of the described reporter mouse system for monitoring inflammatory reactions during the development of inflammatory diseases, we conducted two preclinical models, DSS-induced colitis mouse model and CDAHFD-induced NASH mouse model. First, we subjected NKLL mice to DSS-induced colitis, which has been used to mimic human IBD. Bioluminescence was found to increase over time after DSS supplementation (Fig. 4A). Also, the elevation of luminescence intensity preceded loss of body weight (a symptom of ulcerative colitis) even after 24 h of DSS supplementation (Fig. 4B,C), and a positive correlation was observed between luminescence intensities and DAIs (*r* = 0.813) (Fig. 4D). Furthermore, the development of colitis was confirmed by HE staining of colons (Fig. 4E). These results indicate that observed increases in luminescence were due to DSS-induced colitis.

Next, we applied the CDAHFD-induced NASH model to NKL and NKLA mice to determine whether our reporter mouse could be used to detect NASH non-invasively. Bioluminescence from livers was brighter in CDAHFD treated mice than that in chow diet treated mice, and luminescence intensity was much greater for NKLA mice than for NKL mice (Fig. 5A). On the other hand, we did not observe any significant difference in serum AST levels, which are used as a biomarker of liver injury and body weight and histopathological changes (Fig. 5B–D). Non-alcoholic fatty liver disease (NAFLD) activity scores (NAS) have been used to differentiate healthy individuals and those with NAFLD or NASH and can be applied to rodent NASH models. After two weeks of CDAHFD challenge, NAS, steatosis, and inflammatory scores were increased. However, no differences were observed between genotypes (Fig. 5E), and these histopathological scores were positively correlated with liver luminescence intensities in reporter mice (Fig. 5F). In particular, since bioluminescence signals from NKLA mice were much stronger than those from NKL mice, NKLA mice were considered to be more useful for the non-invasive monitoring of liver diseases.

Taken together, our novel BLI-based reporter mouse can be utilized for the whole body and cell-type specific non-invasive monitoring of superficial and deep inflammatory diseases and that this reporter system could aid anti-inflammatory drug development.

## Supplementary Information

Supplementary Information.

## Data Availability

Relevant data are available from the corresponding author (S.H.O.) upon receipt of reasonable request.
