# Supplementary material for: Novel NF-κB reporter mouse for the non-invasive monitoring of inflammatory diseases
Source: Sci Rep. 2023 Mar 2;13:3556. doi: 10.1038/s41598-023-29689-4 (PMC9981691; doi:10.1038/s41598-023-29689-4)

**Novel NF-κB Reporter Mouse for the Non-invasive Monitoring of Inflammatory Diseases**

Se Yong Park^1,†^, Min Woo Kim^2,†^, Ju-Hee Kang^3^, Hyun Jin Jung^3^, Jung Ho Hwang^3^, Soo Jung Yang^3^, Jong Kyu Woo^4^, Yoon Jeon^5^, Ho Lee^5^, Yeo Sung Yoon^1^, Je Kyung Seong^4*^, Seung Hyun Oh^3*^

^1^ College of Veterinary Medicine, Seoul National University, Seoul, Republic of Korea

^2^ College of Veterinary Medicine, Konkuk University, Seoul, Republic of Korea

^3^ College of Pharmacy, Gachon University, Incheon, Republic of Korea

^4^ Korea Mouse Phenotyping Center (KMPC), College of Veterinary Medicine, Seoul National University, Seoul, Republic of Korea

^5^ Graduate School of Cancer Science and Policy, Research Institute, National Cancer Center, Goyang, Republic of Korea

^†^ These authors contributed equally

**^*^ Corresponding authors**

**Je Kyung Seong (snumouse@snu.ac.kr)**

Korea Mouse Phenotyping Center (KMPC), Seoul National University, Seoul, Republic of Korea

**Seung Hyun Oh (eyeball@hanmail.net, eyeball@gachon.ac.kr)**

College of Pharmacy, Gachon University, Incheon, Republic of Korea

**Primer sequences used for genotyping**

The primers used to distinguish wildtype (603 bp) and mutant (329 bp) ROSA26 allele were as follows; ROSA26 forward (Common): 5'-AAA GTC GCT CTG AGT TGT TAT-3', ROSA26 reverse (WT): 5'-GGA GCG GGA GAA ATG GAT ATG-3', ROSA26 reverse (Mut): 5'-GGC GGG CCA TTT ACC GTA AG-3'. The primers used for Lyz2-Cre genotyping (WT ~350 bp, Mut ~700 bp) were IMR3066 (Mut): 5'-CCC AGA AAT GCC AGA TTA CG-3', IMR3067 (Common): 5'-CTT GGG CTG CCA GAA TTT CTC-3', and IMR3068 (WT): 5'-TTA CAG TCG GCC AGG CTG AC-3'. The primers used for Alb-Cre genotyping (Mut ~ 100 bp) were IMR1084: 5'-GCG GTC TGG CAG TAA AAA CTA TC-3', and IMR1085: 5'-GTG AAA CAG CAT TGC TGT CAC TT-3'.

**Primary mouse macrophages isolation and culture**

L929 cells were purchased from ATCC (Manassas, VA, USA) and cultured in Roswell Park Memorial Institute (RPMI) 1640 medium (Welgene, Daegu, Korea) supplemented with 10% fetal bovine serum (FBS) (Welgene) and 1% penicillin/streptomycin (Welgene). L929 conditioned medium was prepared by seeding L929 cells on 100 mm cell culture dishes at 4.5 × 10^6^ cells/dish in 7 ml of RPMI 1640 medium. Five days later, culture media were collected and stored at -20℃ until used to induce monocyte to macrophage differentiation. Under anesthesia, femurs were extracted from 8–10 weeks old mice, and bone marrows was incubated in RPMI1640 medium containing 20% L292 conditioned medium. Five days later, adherent cells (bone marrow-derived macrophages; BMDMs) were seeded into 6 or 96 well cell culture plates.

Peritoneal macrophages were collected from peritoneal cavity by flushing three times with PBS, incubated for 2 h at 37°C in cell culture plates, and then non-adherent cells were removed by washing. Adherent cells (peritoneal macrophages) were used for further experiments.

BMDMs or peritoneal macrophages were treated with LPS or BAY 11-7085 (an NF-κB inhibitor) (Cayman Chemical, MI, USA), as described in figure 3.

**Primary mouse hepatocyte isolation and culture**

Eight to ten weeks old NF-κB-Luc (NKL) and NF-κB-Luc:Alb (NKLA) mice were anesthetized by isoflurane inhalation. Inferior vena cavae were cannulated using a 24-gauge needle, and portal veins dissected. Livers were perfused with perfusion buffer [142 mM NaCl, 6.7 mM KCl, 10 mM HEPES, 0.095% (w/v) EGTA, pH 7.4] at a rate of 8.49 ml/min for 6 min at 37℃, and then with digestion buffer [66.7 mM NaCl, 6.7 mM KCl, 50 mM HEPES, 4.8 mM CaCl·2H_2_O, pH 7.6 containing 0.075% (w/v) of collagenase type I (Worthington Biochemical Corporation, Lakewood, NJ, USA) and 0.42% (w/v) of fatty acid free bovine serum albumin (BSA; GenDEPOT, TX, USA)] at a rate of 8.49 ml/min for 6 min at 37℃, with intermittent portal vein obstruction. Perfused livers were transferred to Petri dishes containing 15 ml of primary hepatocyte culture medium [low glucose DMEM (Welgene) containing 10% (v/v) FBS (Welgene), 1% penicillin/streptomycin (Welgene), 23 mM HEPES, and 40 ng/ml dexamethasone] and mechanically dissociated. Liver suspensions were passed through a 70 μm pore strainer (BD, CA, USA) and centrifuged at 50 × g for 4 min at 4°C. Cell pellets were gently washed with primary hepatocyte culture medium and centrifuged at 50 × g for 4 min at 4°C, and the pellets obtained were resuspended in primary hepatocyte culture medium, placed carefully on 42% (v/v) Percoll (GE Healthcare, Uppsala, Sweden) solution, and centrifuged at 340 × g for 5 min at 4°C. Pellets of primary hepatocytes below the Percoll solution layer were collected and seeded into Corning Primaria™ 6-well plates (Corning, NY, USA) or 96-well cell culture plates. Media were replaced with fresh media 4 h after plating, and adherent cells were treated with recombinant murine TNFα (Peprotech, NJ, USA) or BAY 11-7085 as described in figure 3.


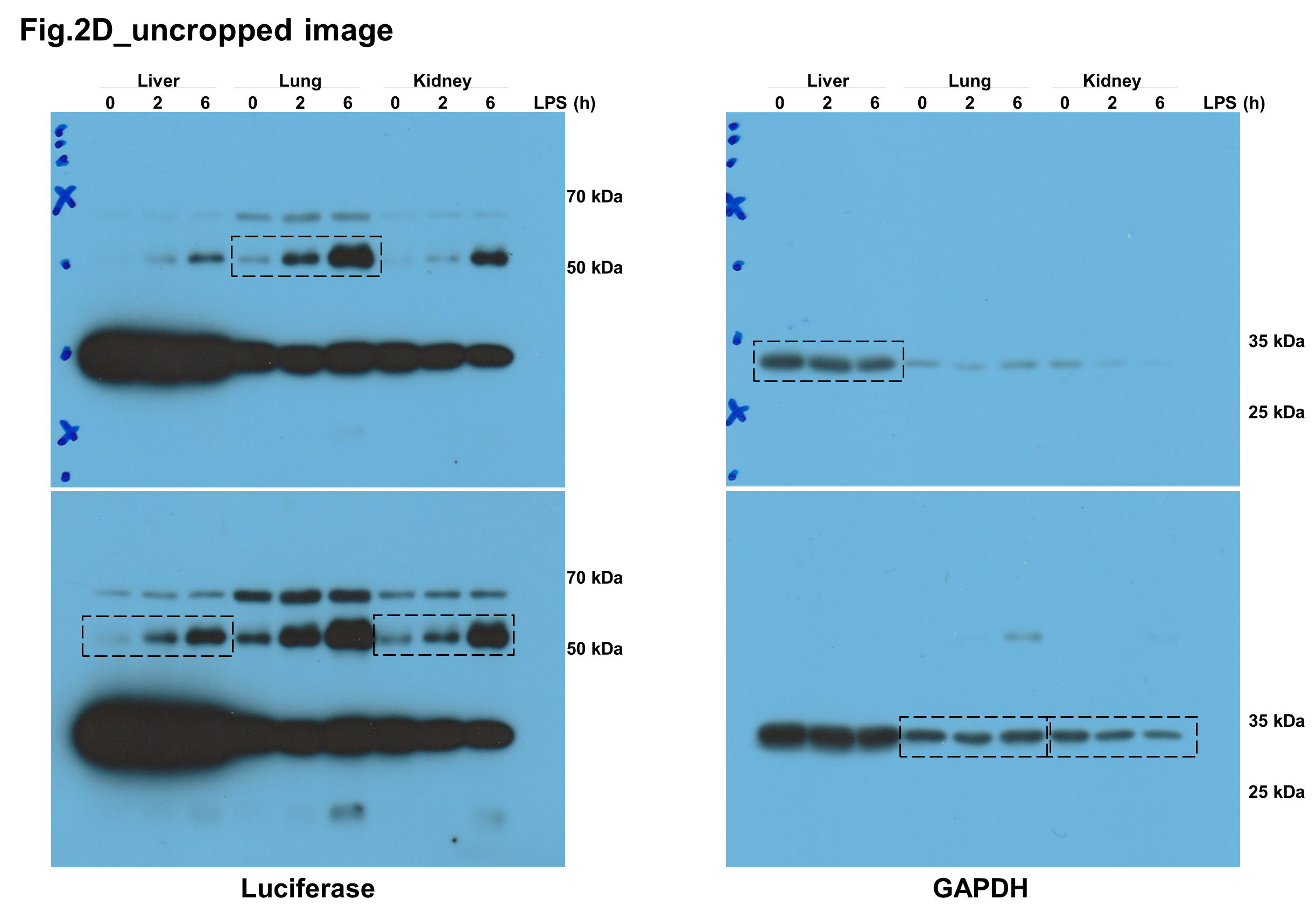


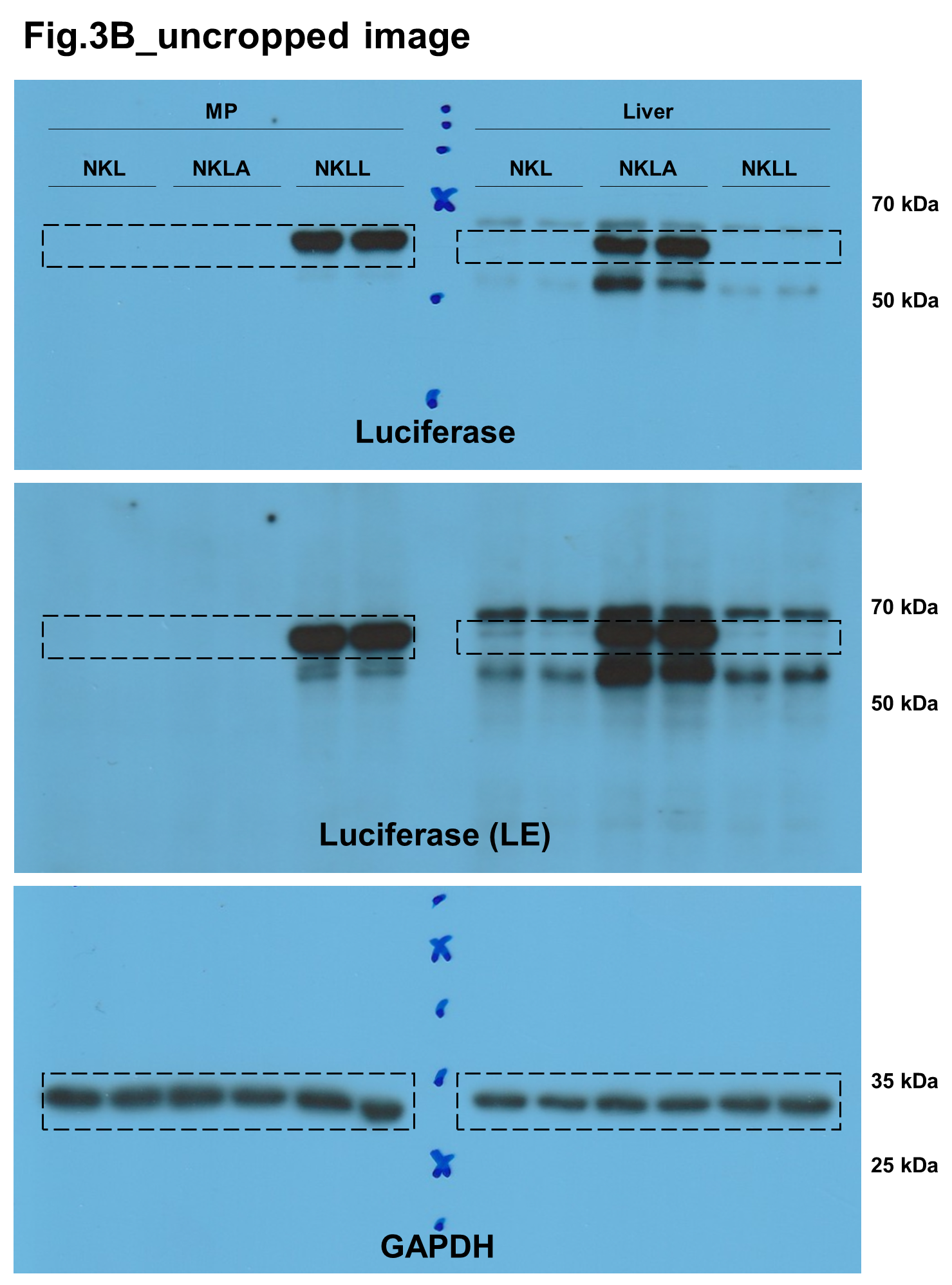


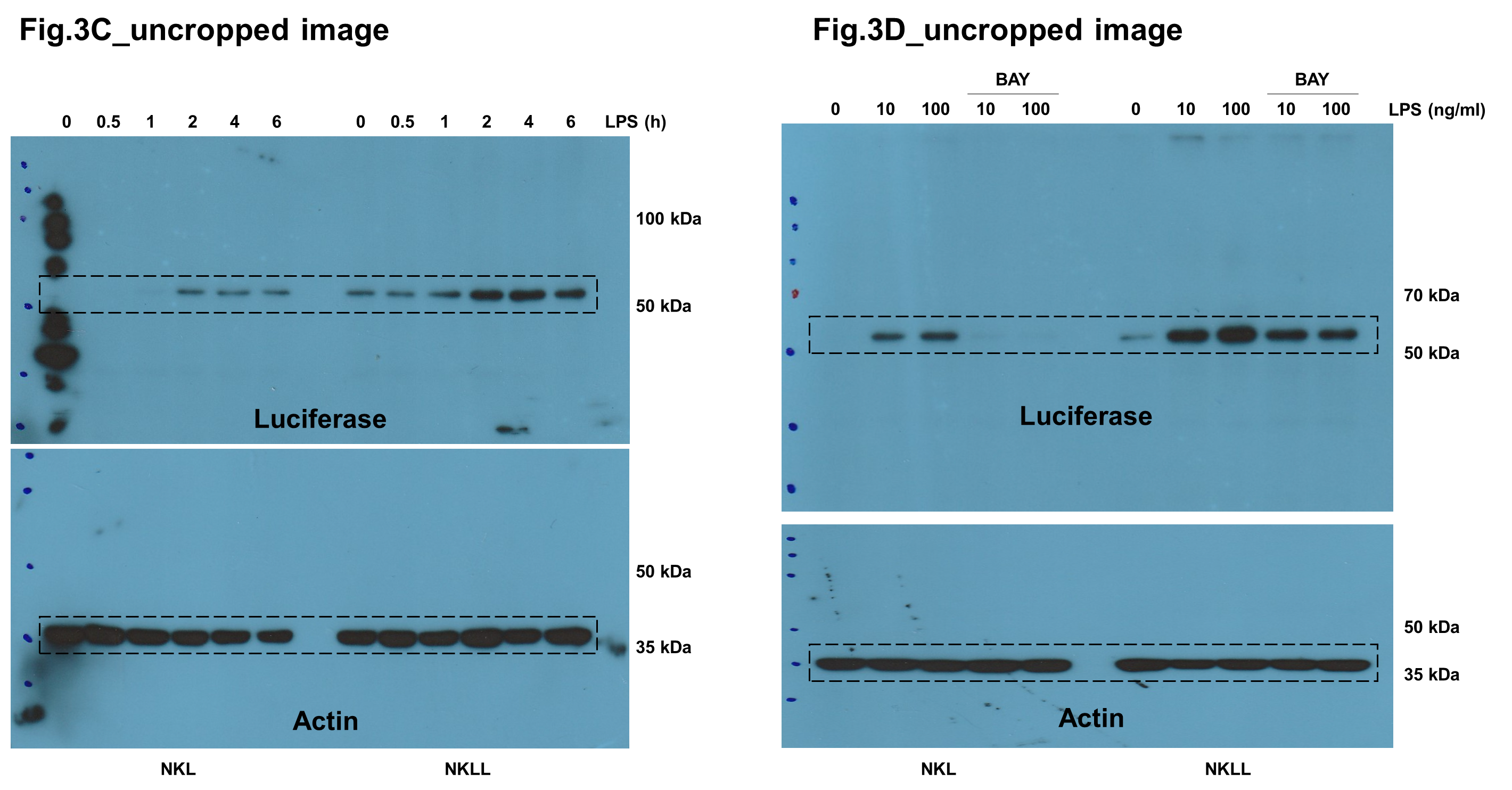

Supplement: Supplementary file 1 — Supplementary Information. [file 41598_2023_29689_MOESM1_ESM.docx]
